# Systemic Therapy of Neuroendocrine Neoplasia: Single Center Experience from a Cohort of 110 Consecutive Cases

**DOI:** 10.1155/2020/1491475

**Published:** 2020-01-31

**Authors:** Karin Mayer, Selina Kiry, Anna Yordanova, Hojjat Ahmadzadehfar, Florian C. Gaertner, Ralph A. Bundschuh, Markus Essler, Maria A. Gonzalez-Carmona, Christian P. Strassburg, Hanno Matthaei, Philipp Lingohr, Savita Bisht, Peter Brossart, Georg Feldmann

**Affiliations:** ^1^Department of Internal Medicine 3, Center of Integrated Oncology Aachen-Bonn-Cologne-Duesseldorf, University Hospital of Bonn, Bonn, Germany; ^2^Department of Nuclear Medicine, Center of Integrated Oncology Aachen-Bonn-Cologne-Duesseldorf, University Hospital of Bonn, Bonn, Germany; ^3^Department of Internal Medicine 1, Center of Integrated Oncology Aachen-Bonn-Cologne-Duesseldorf, University Hospital of Bonn, Bonn, Germany; ^4^Department of Surgery, Center of Integrated Oncology Aachen-Bonn-Cologne-Duesseldorf, University Hospital of Bonn, Bonn, Germany

## Abstract

**Objective:**

Neuroendocrine neoplasias (NENs) represent a rare and biologically heterogeneous group of malignancies. Treatment of NEN patients remains challenging due to lack of prospective evidence on the choice of ideal therapeutic sequence and therapeutic efficacy in specific individual scenarios.

**Methods:**

Clinical data on 110 consecutive patients suffering from NEN treated at a single German university center were analyzed, therapeutic regimens applied were assessed, and the outcome was evaluated.

**Results:**

Histological grading, Ki67 proliferation index, functional activity, and presence of metastases were identified as prognostic markers. 10-year overall survival rates were 92%, 44%, and 0% for G1, G2, and G3 tumors, and 60%, 39%, 69%, 53%, and 0% for Ki67 <2%, 3–5%, 6–20%, 21–49%, and >50%, respectively. Peptide receptor radionuclide therapy (PRRT) and cytostatic chemotherapy were the second most common options, with PRRT being used more frequently in NET G1 and G2 and chemotherapy in NEC G3. Combination chemotherapy with etoposide plus cisplatin or carboplatin showed disease control rates (DCRs) of overall 74%, with a short median progression-free survival (PFS) of 7 or 5 months, respectively. DCR and PFS for PRRT were 89% and 22 months when administered as monotherapy, versus 100% and 27 months upon combination with somatostatin analog (SSA) therapy. Of note, PRRT also achieved disease control as best response in 5/5 (100%) selected cases of NEC G3.

**Conclusion:**

Further prospective studies are warranted to help stratify available options for therapeutic intervention in NEN patients.

## 1. Introduction

Neuroendocrine neoplasms (NENs) represent a rare, biologically highly diverse group of human malignancies with overall rising incidence rates [1]. In line with this biological heterogeneity, overall prognosis and response to therapeutic intervention vary widely among NEN patients. Recent years have seen significant improvements in clinically available diagnostic tools as well as therapeutic options. Namely, there is now a considerable portfolio of locally ablative approaches such as novel cytoreductive surgical techniques, radiotherapy, selective internal radiation therapy (SIRT), and focused radiotherapy as well as systemic therapeutic options, including biotherapy with interferons or somatostatin analogs (SSA), peptide receptor radionuclide therapy (PRRT), classical cytostatic chemotherapy, tyrosine kinase inhibitors as well as novel molecularly targeted approaches comprising antibodies or small molecule inhibitors among others [2–4].

On the other hand, due to a substantial lack of controlled prospective data, the optimal choice of a therapeutic regimen in a given patient is often difficult and there is uncertainty as to the optimal sequence of therapeutic lines in different scenarios [5]. As of to date, therapeutic sequences for individual patients are ideally agreed upon after thorough interdisciplinary discussion in the form of standardized tumor board meetings, which have been shown to improve patient outcome [6, 7], and these decisions are necessarily strongly influenced by personal experience and preferences of tumor board members involved in these discussions.

Here, a series of 110 consecutive neuroendocrine tumor patients treated at a single center in Germany is reported and therapeutic regimens applied are analyzed.

## 2. Materials and Methods

### 2.1. Patients

Medical records of 110 patients suffering from neuroendocrine neoplasia (NET G1-G2 or NEC G3) treated at the University Hospital of Bonn, Germany, between January 2005 and December 2015 were analyzed retrospectively. All available written paper documents as well as electronic patient records were included in this analysis.

Relevant clinical data including age, gender, disease-related symptoms as well as tumor-specific characteristics including date of the first diagnosis, primary tumor localization, histological grading (according to WHO 2017 classification), proliferation index as well as relevant laboratory parameters were entered into an electronic database following predefined criteria.

### 2.2. Systemic Therapy

All therapeutic decisions were made following established in-house standard operating procedures, and cases were discussed routinely in an interdisciplinary tumor board.

Biotherapy with somatostatin analogs (SSA), systemic tyrosine kinase inhibitor therapy, and administration of cytostatic chemotherapy were carried out in line with established guidelines [6, 8, 9]. Administration of peptide receptor radionuclide therapy (PRRT) and response assessment was performed as previously described [10]. Routine follow-up was performed usually in intervals of three to six months. In addition to routine physical and laboratory follow-up including assessment of neuron-specific enolase (NSE) and chromogranin A (CgA) as tumor markers, restaging was performed using standard-of-care imaging including magnetic resonance tomography (MRT) or computed tomography (CT) scans, supplemented by Ga-68-DOTATOC-PET/CT or, in some cases with G3 histology, fluorodeoxyglucose (FDG)-PET/CT following interdisciplinary tumor board consensus recommendations for each individual case. Response to therapy was documented following standard RECIST criteria [11] and documented as complete (CR), partial (PR) or minimal response (MR), stable disease (SD), or progressive disease (PD). For each individual line of systemic therapy, the best therapeutic response and time to progression were documented for every case.

### 2.3. Statistical Analysis

Data for statistical evaluation were collected in an electronic database as described above. Fisher's exact test, chi-square test, and Kaplan–Meier analyses were performed using IBM SPSS version 22.0 for MS Windows. *p* < 0.05 was considered statistically significant.

## 3. Results

### 3.1. Patient Characteristics

A total of *n* = 110 cases of neuroendocrine neoplasms (NENs) were included in this analysis, of these 56 (51%) were female and 54 (49%) were male. The mean age at the time of first diagnosis was 58.2 years, with a wide range from 14 to 83 years (Table 1). Roughly two-thirds (68.2%) of cases comprised neuroendocrine tumors with a histological grading of G1 or G2, while 26 cases (23.6%) were classified as G3 neuroendocrine carcinomas. 21 (19.1%) of 110 cases were considered functional NEN, presenting with isolated flush (8/110; 7.3%), diarrhea (2/110; 1.8%), or concomitant flush and diarrhea (11/110; 10%), while 80.9% (89/110) of cases were found to be nonfunctional. The vast majority of patients presented with favorable performance status of ECOG = 0 (75.5%) or 1 (16.4%).

**Figure 1:**
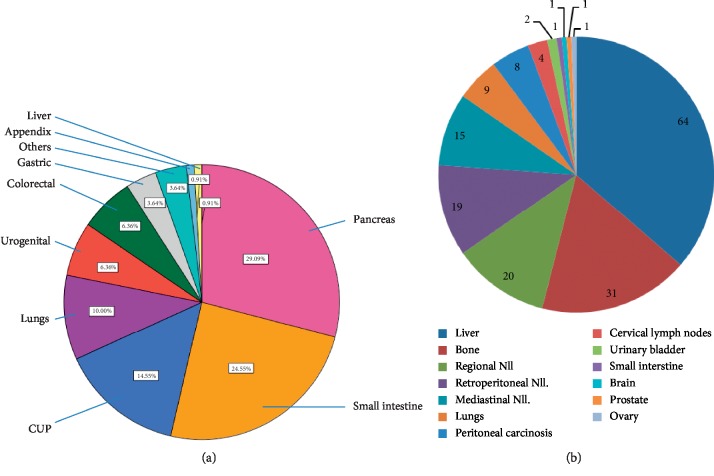
Distribution of primary tumor localizations (a) and sites of distant metastases (b) at the time of initial diagnosis.

The most common localizations of primary tumors were in the pancreas with 32 (29.1%) of 110 cases or small intestine with 27 (24.6%) of 110, while 16 (14.6%) of 110 cases were considered cancers with unknown primary (CUP) (Figure 1(a)). Neuroendocrine neoplasias of the lung represented 10% (11/110) of cases evaluated here. At the time of first diagnosis, 85 (77.3%) of 110 patients presented with systemic metastases, most commonly to the liver (64/110; 58.2%), regional or distant lymph nodes (46/110; 41.8%), bones (31/110; 28.2%), lung (9/110; 8.2%), or peritoneum (8/110; 7.3%) (Figure 1(b)).

### 3.2. Prognostic Markers

**Figure 2:**
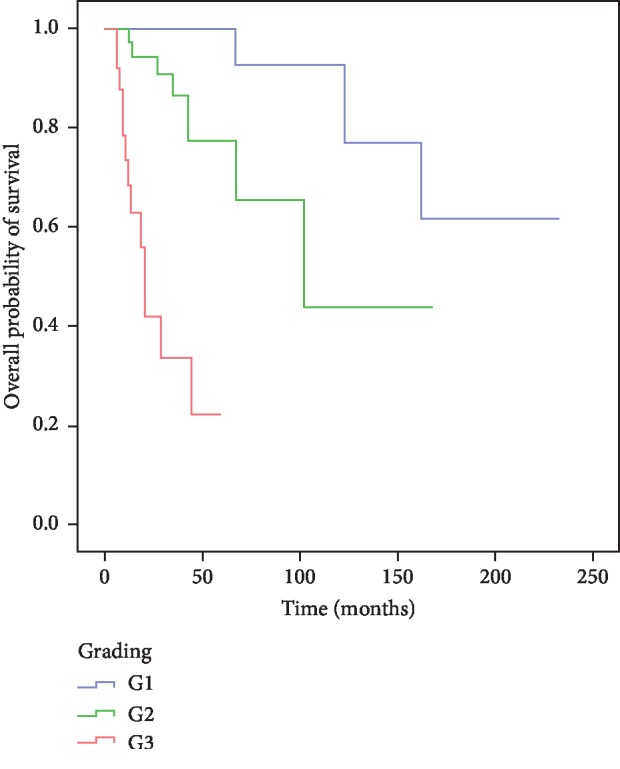
Kaplan–Meier analysis of overall survival of patients suffering from NET G1 (*n* = 35; blue), G2 (*n* = 40; green), or NEC G3 (*n* = 26; red).

Gender and age below or above 60 years at the time of initial diagnosis were not found to have an effect on overall survival (data not shown). Of note, and in line with previous reports by others, histological grading was a strong predictor of overall patient survival in the cohort described here (Figure 2). Overall survival differed widely between G1, G2, and G3 tumors (*p* < 0.01) and median overall survival was not reached in the cohort of G1 tumors, while it was 102 months for histological grade G2 and 20 months for G3. Likewise, this difference in prognosis was mirrored strikingly in the observed 5-year survival rates of 100%, 77%, and 22%, and 10-year survival rates of 92%, 44%, and 0% for NEN with histological grades G1, G2, or G3, respectively.

**Figure 3:**
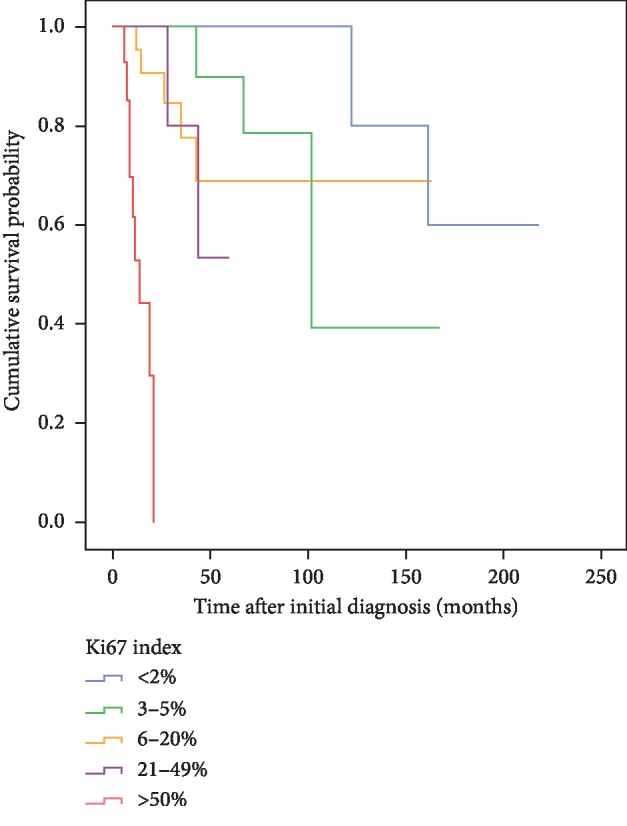
Kaplan–Meier analysis of overall survival depending on proliferation indices of primary tumors, Ki67 <2% (*n* = 28; blue), Ki67 3–5% (*n* = 16; green), Ki67 6–20% (*n* = 24; yellow), Ki67 21–49% (*n* = 10; pink), and Ki67 >50% (*n* = 14; red).

Similarly, proliferation index determined at the time of initial diagnosis predicted overall survival. Estimated median overall survival was 102 months for the group of patients carrying tumors with Ki67 indices between 3 and 5%, but only 13 months for the subgroup with Ki67 >50%, while median overall survival was not reached for the other cohorts analyzed, probably due to limited sample size for each respective cohort. The subgroup of patients with NEN showing Ki67 proliferation indices of <2%, 3–5%, 6–20%, 21–49%, or >50% had estimated 5-year overall survival rates of 100%, 90%, 69%, 53%, and 0%, while 10-year survival rates were 60%, 39%, 69%, 53%, and 0%, respectively (Figure 3).

**Figure 4:**
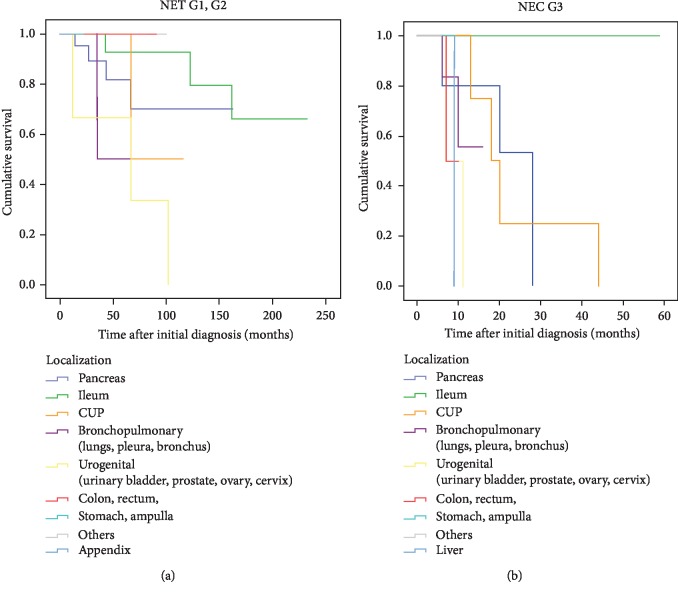
Adverse prognosis of NEC G3 as compared to NET G1 or G2 originating from different primary tumor localizations (Kaplan–Meier analysis of overall survival).

Most likely due to the relatively small number of subjects per cohort, a significant difference in overall survival depending on primary tumor localization could not be detected; there appeared to be a trend for particularly poor prognosis of Pan-NET (G1 and G2) as compared to NET originating from other sites (Figure 4(a)), and NEC G3 apparently carried worse overall prognoses in each localization as compared to NET G1 and G2 counterparts (Figure 4).

Functionally inactive NEN seemed to carry an adverse prognosis in terms of overall survival as compared to functionally active counterparts (data not shown), possibly due to an overall lack of specific early symptoms in nonfunctional NEN, often rendering correct diagnosis difficult. Unsurprisingly, presence of systemic metastases represented an adverse prognostic factor, irrespective of histological grade or proliferative capacity (Supplemental [Supplementary-material supplementary-material-1] and Supplemental [Supplementary-material supplementary-material-1]).

### 3.3. First-Line Therapy Options

Surgical resection was the most commonly applied first-line therapeutic option in the cohort studied here. In 11 (10%) of 110 cases, surgery was accompanied by immediate initiation of SSA therapy, and 4 of 110 patients received adjuvant chemotherapy in combination with surgical resection, while upfront surgical resection alone without any form of concomitant systemic therapy was the modality of choice in 47 (43%) of 110 cases. For neuroendocrine tumors with histological grading of G1 or G2, surgical resection was the first-line therapy of choice in nearly two-thirds (44/75 (59%)) of cases, in 10 cases (13%) out of these, SSA therapy was initiated immediately after surgical resection, while in the other 34 cases, surgical resection was not combined with any systemic therapeutic regimen (Table 2). In neuroendocrine carcinomas (NECs) with histological grade G3, surgical resection as first-line therapy was performed in 11 (42%) of 26 documented cases; here, resection was followed by subsequent systemic chemotherapy in 4 of these 11 cases, while in the remaining 7 cases surgery alone without any concomitant systemic therapy was performed.

Systemic chemotherapy and PRRT were the second most commonly used first-line options with respect to the cohort consisting of NEN with histological grades ranging from G1 to G3, administered in 17% (19/110) and 19% (21/110) of cases, respectively. As expected, NEC G3 patients were more likely to receive chemotherapy as first-line regimen as compared to NET G1 or G2 (42% (11/26) versus 11% (8/75) (*p* < 0.01)). Vice versa and not surprisingly, first-line therapy with PRRT was the more commonly picked option in NET G1 or G2 (23% (17/75)), while given in only 2 (8%) out of 26 cases with NEC G3 (NS).

Of interest, SSA monotherapy or watch and wait were relatively rare choices for first-line treatment in the cohort analyzed here, applied in only 7 (6%) and 1 (1%) of 110 cases.

### 3.4. Systemic Therapy: Chemotherapy

During the course of the disease, a total of 42 (38%) of 110 NEN patients studied here received at least one line of chemotherapy, and in 19 of these, chemotherapy was applied as first-line therapy. Direct correlations were observed between the probability of receiving chemotherapy at some point as part of sequential therapy and histological grading as well as Ki67 proliferation index, respectively. While almost all (92%) NEC G3 tumors were treated by means of cytostatic chemotherapy, this fraction was only 35% in NET G2, and with only 2% posed a rare exception in cases with NET G1. Likewise, the fractions of patients receiving cytostatic chemotherapy were 100% (14/14) for tumors with Ki67 proliferation indices above 50%, and 80% (8/10) for Ki67 above 20% but lower than 50%, but were found to be much smaller for more slowly growing malignancies: while approximately half (46%; 11/24) of all patients carrying NET with Ki67 proliferation indices between 6 and 20% received chemotherapy as part of their therapeutic regimens at some point, these numbers dropped to three (19%) of 16 cases for Ki67 between 3 and 5% and two (7%) of 28 cases for Ki67 below 2% (Supplemental [Supplementary-material supplementary-material-1]).

### 3.5. Chemotherapy Regimens Applied and Therapeutic Efficacy

The most commonly applied chemotherapy regimens in this cohort were carboplatin or cisplatin plus etoposide. In the majority of cases, either of these combinations was given to patients suffering from NEC G3 tumors, only 6 of the total 25 carried NET G2, and these combinations were not used in G1 neuroendocrine tumors (Table 3). Other combinations such as streptozotocin/5-FU or temozolomide plus capecitabine were applied far less frequently and mostly to moderately differentiated G2 neuroendocrine tumors. Although the total number of cases was thus limited, it is noteworthy that the therapeutic efficacy of cisplatin plus etoposide or carboplatin plus etoposide was within the same range, with median progression-free survival of seven or five months, respectively (Supplemental [Supplementary-material supplementary-material-1]). Neither of both combination chemotherapy regimens induced a complete remission. As best therapeutic response, partial remission or stable disease was achieved in 1/5 (20%) or 3/5 (60%) of cases treated with cisplatin plus etoposide, and 8/18 (44%) or 5/18 (28%) for carboplatin plus etoposide; median progression-free survival for carboplatin plus etoposide or cisplatin plus etoposide was five months or seven months, respectively.

### 3.6. Peptide Receptor Radionuclide Therapy (PRRT)

From the cohort of 110 consecutive patients carrying neuroendocrine neoplasias evaluated here, a total of 69 (63%) received at least one line of PRRT as part of their individual sequential course of treatment, either alone or in combination with systemic SSA therapy (Table 4 and Supplemental [Supplementary-material supplementary-material-1]). PRRT was much more frequently administered in well to moderately differentiated NEN: while 73% (55/75) of patients with NET G1 or G2 received at least one line of PRRT, this fraction decreased to 23% (6/26) for NEC G3. Similarly, PRRT was a more common choice of therapy in slower proliferating neoplasias: for Ki67 proliferation indices of up to 20%, the fraction of cases treated with PRRT was 74% (50/68), while these numbers dropped to 50% (5/10) for proliferation indices of up to 50%, and only 1 (7%) of 14 cases with Ki67 above 50% received PRRT. PRRT alone or in combination with SSA therapy was not sufficient to induce a complete response in any of the cases documented here, and disease control rates were 89% (31/35) for PRRT monotherapy and 100% (30/30) for PRRT plus SSA combination. Of interest, median progression-free survival tended to be longer for PRRT plus SSA combination (27 months) as compared to PRRT alone (17 months), underscoring the potential clinical relevance of this combinatorial approach.

Despite the limited number of evaluable cases, it is another noteworthy observation of this analysis that PRRT—either as monotherapy or in the form of PRRT plus SSA combination—enabled disease control with at least stable disease as best response in all (100%) five cases with high grade (G3) neuroendocrine carcinomas with a median PFS of 13.5 months (mean: 13.25 months, standard deviation: 2.75 months), suggesting that this currently difficult-to-treat subgroup of patients might benefit from PRRT administration as well (Supplemental [Supplementary-material supplementary-material-1]).

## 4. Discussion

Neuroendocrine neoplasias represent a rare and biologically diverse group of malignancies [12, 13]. Traditionally, the treatment of neuroendocrine neoplasia has primarily been a domain of surgical resection. However, over the past decades, a plethora of locally ablative techniques, such as radiofrequency ablation (RFA), selective internal radiotherapy (SIRT), cytoreductive surgery, as well as systemic therapeutic options, including biotherapy with interferons or somatostatin agonists (SSA), cytostatic chemotherapy, peptide receptor radionuclide therapy (PRRT), tyrosine kinase inhibitors, and other novel molecularly targeted therapies emerged and found their way into routine clinical application [2]. This abundance of therapeutic options is currently accompanied by a relative lack of experimental data on the optimal sequence of therapeutic strategies to be applied in each individual NEN case [5]. Therefore, all therapeutic decisions affecting NEN patients should today be discussed in an interdisciplinary tumor board including representatives of all major disciplines involved, namely, pathology, medical oncology, surgery, radiology, radiotherapy, nuclear medicine, and gastroenterology [6, 7]. Of interest, treatment of these relatively rare malignancies in specialized centers and standardized routine tumor board discussions appear to have the potential to enhance the therapeutic success and overall patient outcome [14, 15].

Several landmark studies from recent years began to uncover underlying genomic alterations driving tumor initiation and progression of neuroendocrine neoplasia. As opposed to other human malignant solid tumors, such as pancreatic, lung, or colorectal carcinomas, somatic oncogenic driver mutations were found to be relatively rare in neuroendocrine tumors of pancreatic or small intestinal origin [16, 17]. More recently, recurrent driver mutations have been identified in subsets of NEN, opening up potential avenues for targeted therapeutic intervention in the future. Mutations in MEN1 were found in 44% and DAAX or ATRX in 43% and alterations affecting the mTOR signaling pathway were identified in 15% of cases studied [18]. Of interest, this study also described oncogenic mutations in pathways affecting DNA damage response, including MUTYH in 6%, CHEK2 in 4%, and BRCA2 in 1% of cases. Mutations in Kras or TP53, which are frequently detected in ductal adenocarcinoma of the pancreas, were significantly less frequently observed in pancreatic neuroendocrine neoplasia (PanNEN) [19]. Despite these encouraging findings, specific targeted therapeutic intervention based on individual thorough genomic evaluation is not the norm for the majority of NEN patients treated today, and clinically relevant prospective data on the best sequence of availably therapeutic options in individual cases and therapeutic efficacy of available systemic therapeutic options are scarce [5]. Therefore, more basic clinical and pathological parameters are usually more relevant in determining therapeutic strategies in NEN, such as histological tumor grading, tumor stage, and growth rate as well as patient-immanent factors such as age and comorbidities [6–9]. In this report, clinically relevant prognostic factors, therapeutic regimens applied, and response to systemic therapy were evaluated in a cohort of 110 neuroendocrine neoplasia patients treated at a single university center.

Of interest, our data clearly support the tremendous prognostic significance of histological grading and Ki67 proliferation index. These observations are in line with previous reports by others and support the central role of these parameters in current classification systems and clinical guidelines [6–9]. The relatively low 10-year survival rate for Ki67 of 3–5% found here might reflect a sampling error due to the small number of individuals included in this subcohort.

Of note, in this analysis, there was no difference in patient outcome for age groups <60 years as compared to >60 years at the time of initial diagnosis. This observation is in line with two previously published reports by others [20, 21], suggesting that advanced age alone should not generally preclude patients from receiving adequate therapeutic intervention, but rather discussed in an interdisciplinary setting on an individualized case-by-case basis.

It has long been discussed that tissue of origin and primary tumor localization are highly relevant with respect to metastatic potential and overall prognosis of NEN and should therefore also be considered for individualized choice of a preferred therapeutic sequence [2]. Our data presented here did not support this hypothesis, and major differences in overall survival could not be observed in NET G1 or G2 depending on the primary tumor localization, possibly due to the small number of cases in each subgroup. Of note, and in line with previous reports by others [5], it was also found that for specific primary tumor localizations, NEC G3 frequently showed significantly worse prognosis and overall survival as compared to NET G1 or G2. Moreover, despite the relatively small number of individuals included in this retrospective analysis, our data confirmed a strong prognostic value of histological grading and Ki67 proliferation index irrespective of other factors such as primary tumor localization, which is in line with previous observations made by others [22–24].

The rate of SSA applied as first-line systemic therapeutic option is surprisingly low in the cohort described here, likely reflecting a selection bias since patients presenting at our university center might tend to carry overall more advanced disease stages and tumor burden with comparatively high therapeutic urgency.

Prospective data on the therapeutic efficacy of systemic chemotherapy in neuroendocrine neoplasms is widely lacking, and available efficacy data are mainly based on small retrospective analyses [25–27]. In our cohort reported here, a total of 25 patients received combination chemotherapy with cisplatin or carboplatin in combination with etoposide as part of their sequential therapeutic regimen, the majority of cases representing NEC G3. Possibly due to the lack of current reports on larger patients cohorts, the range of reported therapeutic efficacy of these regimens in terms of progression-free survival, disease control rates (DCR), and objective response rates (ORR) is very wide. However, realistically estimating therapeutic efficacy is crucial when choosing a therapeutic regimen over other alternatives in a given patient and for obtaining a patient's informed consent. In this present analysis, we observed ORR of 39% and DCR of 74% for cisplatin or carboplatin plus etoposide combination chemotherapy, with median PFS in the range of 5–7 months, which is well in line with previously reported cohorts [27].

Recent evidence established PRRT as a safe systemic therapeutic option in neuroendocrine tumors with the capacity to delay tumor progression and to enhance overall survival [28, 29]. Following favorable results of the prospective NETTER-1 trial, PRRT should now generally be considered as potential second-line therapeutic option after failure of biotherapy in advanced intestinal neuroendocrine tumors or after the failure of tyrosine kinase inhibitor or chemotherapy in Pan-NET [7, 28, 30]. In our cohort, PRRT was chosen in a considerable fraction of 73% of all neuroendocrine tumor cases evaluated. Of interest, PRRT was also administered in NEC G3 in 23% of cases, an option that is currently underrepresented in recent consensus guidelines [6]. Although biotherapy with SSA in neuroendocrine tumor patients might often be administered mainly as a means of symptom control, direct antineoplastic activity of SSA therapy has now also been well documented in the prospective PROMID and CLARINET phase 3 clinical trials [31, 32]. Moreover, it has previously been reported that combination of PRRT with chemotherapy or with biotherapy may enhance therapeutic efficacy and overall survival in suitable subgroups of NEN patients [10, 33]. Our cohort reported here is in line with these findings, and median progression-free survival was enhanced from 22 months for PRRT only to 27 months for PRRT plus SSA combination. It has previously been speculated that this may be due to a need for continuous antiproliferative therapy conferred by SSA in order to stop tumor progression, while PRRT can only be administered in predefined intervals.

## 5. Conclusions

Analysis of this present cohort confirmed that NENs represent a biologically and histologically diverse group of malignancies, primary tumors were most commonly in the pancreas or small intestine, and “cancer of unknown primary” was the third most common designation. Approximately three-quarters of cases in this cohort were diagnosed at advanced, already metastatic tumor stages. Histological grading, Ki67 proliferation index, functional activity, and presence of metastases were identified as predictive markers. Surgery was the most commonly carried out first-line therapeutic option, either alone or in combination with systemic therapy, and PRRT and chemotherapy were the second most common choices, particularly for NET G1, G2, or NEC G3, respectively.

Therapeutic efficacy of cisplatin or carboplatin plus etoposide combination chemotherapy was within the range of previously reported cohort with relatively short PFS. Response to PRRT was more pronounced upon combination with SSA and was a viable option not only for NET G1 and G2 but also in selected cases with NEC G3.

Further prospective studies are urgently required to identify and validate predictive molecular biomarkers and to help inform treating physicians on the best sequential application of available therapeutic options.

## Figures and Tables

**Table 1:** Patients' characteristics.

Parameter	*n* (%)
Gender	
Female	56 (50.9)
Male	54 (49.1)

Age (years) at first diagnosis	
<60	64 (58.2)
>60	46 (41.8)
Range (years) (min-max (mean))	14–83 (58.2)

ECOG performance status	
0	83 (75.5)
1	18 (16.4)
2	3 (2.7)
3	6 (5.5)

Functional activity	
Nonfunctional	89 (80.9)
Isolated flush	8 (7.3)
Isolated diarrhea	2 (1.8)
Concomitant flush and diarrhea	11 (10.0)

Histological grading	
NET G1	35 (31.8)
NET G2	40 (36.4)
NEC G3	26 (23.6)
Missing data	9 (8.2)

**Table 2:** First-line therapy options applied.

Therapeutic regimen	Entire cohort (*n* = 110) *n* (%)	NET G1 and G2 (*n* = 75) *n* (%)	NEC G3 (*n* = 26) *n* (%)
Surgical resection	62 (56)	44 (59)	11 (42)
Without systemic therapy	47 (43)	34 (45)	7 (27)
Followed by SSA	11 (10)	10 (13)	0 (0)
Followed by chemotherapy	4 (4)	0 (0)	4 (15)
Chemotherapy	19 (17)	8 (11)	11 (42)
PRRT	21 (19)	17 (23)	2 (8)
Without systemic therapy	11 (10)	9 (12)	1 (4)
Followed by SSA	10 (9)	8 (11)	1 (4)
SSA	7 (6)	6 (8)	1 (4)
No therapy	1 (1)	0 (0)	1 (4)

**Table 3:** Chemotherapy regimens applied (number of cases; total *n* = 38).

	Total	Histology		
Regimen		G1	G2	G3
Carboplatin/etoposide	18	0	2	16
Cisplatin/etoposide	7	0	4	3
Streptozotocin/5-FU	3	0	2	1
Temozolomide/capecitabine	2	0	2	0
Temozolomide	2	0	1	1
Carboplatin/irinotecan	1	0	0	1
FOLFOX	1	1	0	0
FOLFIRI	1	0	0	1
Cyclophosphamide	1	1	0	0
Dacarbazine	1	0	1	0
Gemcitabine	1	0	1	0

**Table 4:** PRRT therapeutic efficacy.

Modality	Best response	Frequency		PFS (mean ± SD (months))	PFS (median (months))
PRRT alone	CR	0/35	0%		
	PR	12/35	34%	28.0 ± 28.2	17
	SD	19/35	54%		
	PD	3/35	9%		
	MR	1/35	3%		

PRRT + SSA	CR	0/30	0%		
	PR	16/30	53%	31.7 ± 18.1	27
	SD	14/30	47%		
	PD	0/30	0%		
	MR	0/30	0%		

## Data Availability

All data used to support the findings of this study are included within the article.

## Abbreviations

NEN: Neuroendocrine neoplasia
NET: Neuroendocrine tumor
NEC: Neuroendocrine carcinoma
PRRT: Peptide receptor radionuclide therapy
SSA: Somatostatin analog
DCR: Disease control rate
OS: Overall survival
PFS: Progression-free survival
SIRT: Selective internal radiation therapy.

## Supplementary Materials

Supplementary Materials Supplemental figure 1: presence of systemic metastases confers adverse prognosis in neuroendocrine neoplasia (Kaplan–Meier analysis of overall survival). Supplemental Table 1: patient survival vs. presence of systemic metastases. Supplemental Table 2: frequency of chemotherapy administration versus proliferation index (*n* = 92). Supplemental Table 3: therapeutic efficacy of platinum-based chemotherapy. Supplemental Table 4: frequency of PRRT versus histological grading or proliferation index. Supplemental Table 5: therapeutic response to PRRT versus histological grade.
