# Supplementary material for: Systemic Therapy of Neuroendocrine Neoplasia: Single Center Experience from a Cohort of 110 Consecutive Cases
Source: Int J Endocrinol. 2020 Jan 31;2020:1491475. doi: 10.1155/2020/1491475 (PMC7013359; doi:10.1155/2020/1491475)
Supplement: Supplementary Materials — Supplemental figure 1: presence of systemic metastases confers adverse prognosis in neuroendocrine neoplasia (Kaplan–Meier analysis of overall survival). Supplemental Table 1: patient survival vs. presence of systemic metastases. Supplemental Table 2: frequency of chemotherapy administration versus proliferation index (n = 92). Supplemental Table 3: therapeutic efficacy of platinum-based chemotherapy. Supplemental Table 4: frequency of PRRT versus histological grading or proliferation index. Supplemental Table 5: therapeutic response to PRRT versus histological grade. [file 1491475.f1.pdf]

Supplemental Figure 1

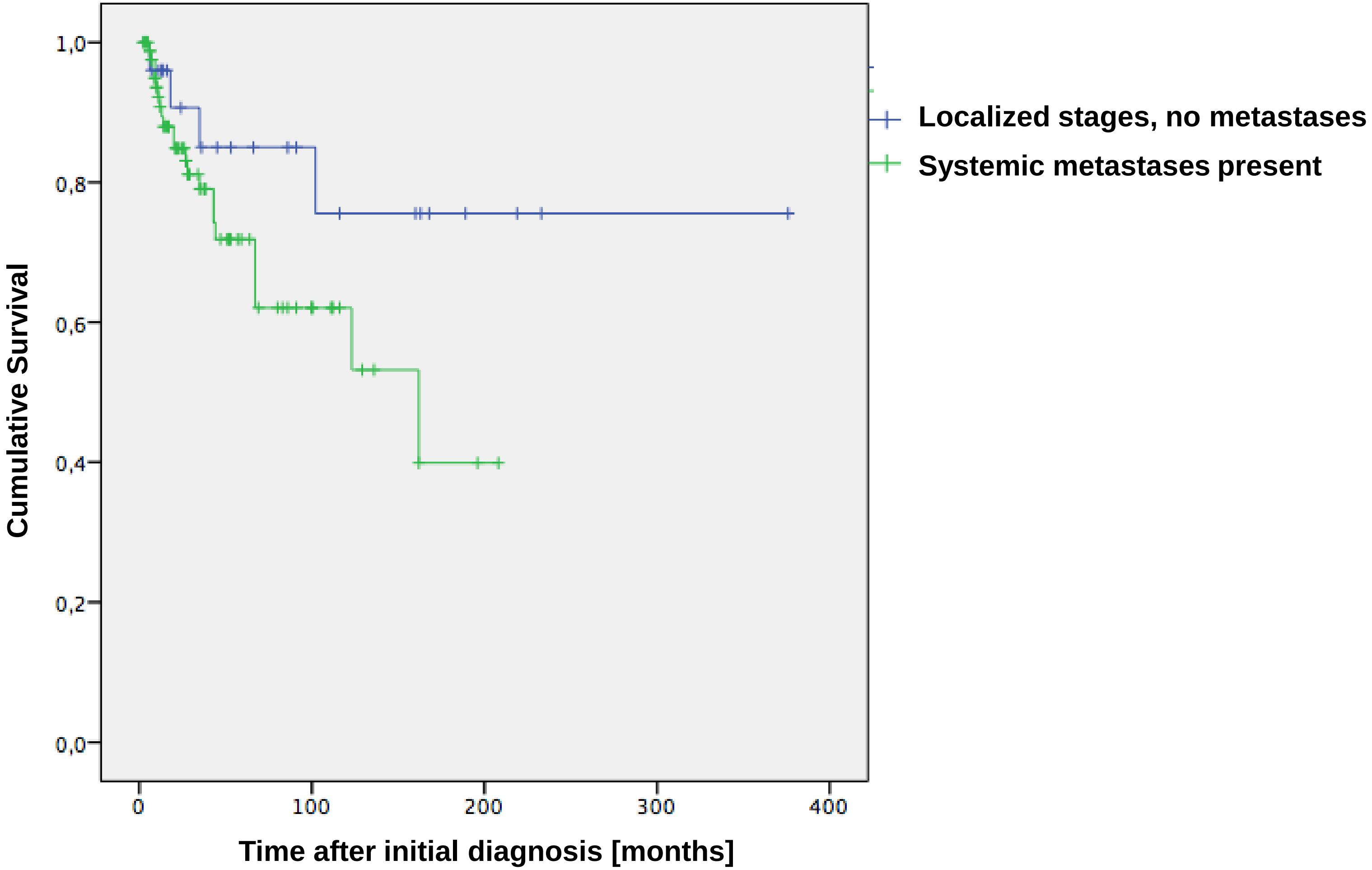

<u>Legend to Supplemental Figure 1:</u>

Presence of systemic metastases confers adverse prognosis in neuroendocrine neoplasia (Kaplan-Meier analysis of overall survival).

Supplemental Table 1:

Patient survival vs. presence of systemic metastases

	**Systemic metastases present** (n=85)	**No metastases** (n=25)
1-year survival rate	85%	95%
5-year survival rate	76%	72%
10-year survival rate	0%	40%

Supplemental Table 2:

Frequency of chemotherapy administration versus proliferation index (n=92).

	Chemotherapy applied	
**Ki67 [%]**	n	(%)
<2	2/28	7
3-5	3/16	19
6-20	11/24	46
21-49	8/10	80
>50	14/14	100

Supplemental Table 3:

Therapeutic efficacy of platinum-based chemotherapy.

	Best response				PFS[1]		
**Regimen**	CR	PR	SD	PD	Median	Mean	SD[2]
Carboplatin/ Etoposid	0	8	5	5	5	7.3	16
Cisplatin/ Etoposid	0	1	3	1	7	5.2	8.9

[1]PFS: Progression-free survival [months]

[2]SD: standard deviation

Supplemental Table 4:

Frequency of PRRT versus histological grading or proliferation index.

Parameter	Fraction treated with PRRT
NET G1 / G2	55/75 (73%)
NEC G3	6/26 (23%)
Ki67<20%	50/68 (74%)
Ki67 21-50%	5/10 (50%)
KI67 >50%	1/14 (7%)

Supplemental Table 5:

Therapeutic response to PRRT versus histological grade.

Histological Grade	CR	PR	SD	PD	MR
G1	0/19 (0%)	6/19 (32%)	11/19 (58%)	2/19 (11%)	0/19 (0%)
G2	0/21	7/21 (33%)	12/21 (57%)	1/21 (5%)	1/21 (5%)
G3	0/5 (0%)	2/5 (40%)	3/5 (60%)	0/5 (0%)	0/5 (0%)
